# The Role of Uterine Natural Killer Cells on Recurrent Miscarriage and Recurrent Implantation Failure: From Pathophysiology to Treatment

**DOI:** 10.3390/biomedicines9101425

**Published:** 2021-10-09

**Authors:** Konstantinos Sfakianoudis, Anna Rapani, Sokratis Grigoriadis, Agni Pantou, Evangelos Maziotis, Georgia Kokkini, Chrysanthi Tsirligkani, Stamatis Bolaris, Konstantinos Nikolettos, Margarita Chronopoulou, Konstantinos Pantos, Mara Simopoulou

**Affiliations:** 1 Centre for Human Reproduction, Genesis Athens Clinic, 14-16, Papanikoli, 15232 Athens, Greece; sfakianosc@yahoo.gr (K.S.); agnipantos@gmail.com (A.P.); margaritagenesis@gmail.com (M.C.); info@pantos.gr (K.P.); 2 Laboratory of Physiology, Medical School, National and Kapodistrian University of Athens, 75, Mikras Asias, 11527 Athens, Greece; rapanianna@gmail.com (A.R.); sokratis-grigoriadis@hotmail.com (S.G.); vagmaziotis@gmail.com (E.M.); ginakokini@gmail.com (G.K.); cheer21295@gmail.com (C.T.); 3 Assisted Conception Unit, Second Department of Obstetrics and Gynecology, Aretaieion Hospital, Medical School, National and Kapodistrian University of Athens, 76, Vasilisis Sofias Avenue, 11528 Athens, Greece; 4 Assisted Conception Unit, General-Maternity District Hospital "Elena Venizelou", Elenas Venizelou Avenue, 11521 Athens, Greece; sbolaris@gmail.com; 5 Assisted Reproduction Unit of Thrace “Embryokosmogenesis”, Apalos, 68132 Alexandroupoli, Greece; k.nikolettos@yahoo.gr

**Keywords:** uterine natural killer cells, assisted reproduction, recurrent implantation failure, recurrent miscarriages, implantation, pregnancy, glucocorticoids, intralipids, intravenous immunoglobulin

## Abstract

Uterine natural killer (uNK) cells constitute a unique uterine leucocyte subpopulation facilitating implantation and maintaining pregnancy. Herein, we critically analyze current evidence regarding the role of uNK cells in the events entailed in recurrent implantation failure (RIF) and recurrent miscarriages (RM). Data suggest an association between RIF and RM with abnormally elevated uNK cells’ numbers, as well as with a defective biological activity leading to cytotoxicity. However, other studies do not concur on these associations. Robust data suggesting a definitive causative relationship between uNK cells and RIF and RM is missing. Considering the possibility of uNK cells involvement on RIF and RM pathophysiology, possible treatments including glucocorticoids, intralipids, and intravenous immunoglobulin administration have been proposed towards addressing uNK related RIF and RM. When considering clinical routine practice, this study indicated that solid evidence is required to report on efficiency and safety of these treatments as there are recommendations that clearly advise against their employment. In conclusion, defining a causative relationship between uNK and RIF–RM pathologies certainly merits investigation. Future studies should serve as a prerequisite prior to proposing the use of uNK as a biomarker or prior to targeting uNK cells for therapeutic purposes addressing RIF and RM.

## 1. Introduction

Natural killer (NK) cells are large granular lymphocytes and have been described as an essential factor of the innate immune system [1]. The cytotoxic ability of NK cells depends on balancing activating and inhibitory signals received from surface receptors [2]. A special category of NK cells localized in uterus are described as uterine natural killer (uNK) cells. During the early pregnancy period, uterine NK (uNK) cells are the largest leukocyte population in the endometrium accounting for over 70% of total endometrial leukocytes [3]. uNK cells significantly differ from the peripheral bloodstream NK cells, since their gene expression program is associated with increased production of cytokines and a relatively low cytotoxic activity. In contrast to peripheral NK cells, uNK cells present a unique pattern of surface markers and are characterized as CD45^+^CD56^bright^CD16^+^CD9^+^ cells [4]. Data provided following a comprehensive transcriptomic analysis employing single-cell RNA-sequencing (scRNA-seq) in tissue samples collected from first-trimester decidua revealed that there are at least three different uNK subpopulations, expressing different patterns of surface markers [5]. This, in turn, leads to the conclusion that these distinct uNK cell subsets exhibit diverse functions and roles [4]. Irrespective of their complicated nature, it is well-established that uNK cells present with increased numbers both in the luteal phase endometrium as well as in early pregnancy decidua [6]. Great focus has been paid to the role of uNK cells in the complex phenomenon of embryo implantation. Contrary to their previously suspected “hostile” characteristics, uNK cells appear to be essential regulators towards achieving successful implantation and pregnancy [7,8]. During pregnancy, uNK cells are involved in numerous crucial physiological events, such as remodeling of the placental vasculature, regulating invading trophoblast cells, and providing immune tolerance.

Embryo quality and endometrial receptivity are the two defining factors that determine successful implantation.

Implantation and establishment of pregnancy represent a dynamic and controversial field of research featuring several pathologies. Recurrent or repeated implantation failure (RIF) is defined as the failure to achieve clinical pregnancy following the transfer of good quality embryos and is diagnosed in the context of ART treatment [9]. Idiopathic RIF refers to the unexplained failure of “cross-talk” between the embryo and the endometrium, which—as previously mentioned—is a prerequisite to ensure apposition, attachment, and penetration of the embryo in the endometrium. Abnormalities may appear in either or both the embryo and endometrium [9] rendering investigation of this phenomenon challenging. Along the same lines of impairing fertility potential, recurrent miscarriage (RM) is another pathological entity sharing common ground with RIF [10]. This distressing condition is diagnosed when the loss of three or more consecutive pregnancies prior to the 20^th^ week of gestation is documented [11,12]. Despite the fact that RM implantation has been achieved, a disruption of the established implantation and failure to sustain the pregnancy will result in a miscarriage. Clearly, both cases of RIF and RM in the context of being encountered by reproduction specialists call for focusing on the investigation of the embryo–endometrium relationship. RIF and RM may emerge during different phases in the embryo’s journey; however, not being able to establish implantation—as evident in RIF—and not being able to sustain the implanted embryo—as evident in RM—may be attributed to shared molecular factors and pathways. Subfertile women present with a higher risk of miscarriages, while in cases of RM patients, if infertility is diagnosed as a coexisting pathology, the prognosis is poor. Therefore, the notion that infertility and RM share overlapping underlying etiological factors has been successfully argued [13,14]. Both implantation failure and miscarriages have been correlated with findings indicating certain abnormalities [10,15,16].

Despite the majority of evidence buttressing the importance of uNK cells in pregnancy, it seems that their excessive presence may impair the reproductive outcome. The implication of uNK cells in implantation and pregnancy maintenance, as well as the association between elevated levels of uNK with RIF and RM [4,5,6,17,18,19], served as the incentive to study in depth the molecular “how” and “why”. Patients diagnosed with RIF or RM experience numerous futile IVF attempts along with psychological distress and financial burden. Therefore, early prognosis markers could be of added value, enabling optimal management options and efficient therapeutic approaches. Could uNK levels serve as such a marker? Could further research indicate that, apart from the association that has been reported, increased uNK levels may lead to RIF and RM? Additionally, if so, through which molecular mechanisms is this realized? Should investigation of these hypotheses prove fruitful, then data could fuel examination on how to address the increased levels as this could further provide a possible therapeutic approach. Data are starting to accumulate on all these aspects; nonetheless, these questions remain technically unanswered. Despite the amount of work in this field, the value of reporting collectively on both RIF and RM is underlined, as both constitute pathologies commonly encountered in ART practice. From the perspective of an ART expert, the lack of an efficient and established therapeutic protocol for RIF and RM patients presents a challenge. Attempting to understand and address RIF and RM pathologies through the prism of uNK cells both diagnostically and therapeutically served as the driver for this study. The purpose of this review is to delineate the molecular events connecting uNK cells to RIF and RM. Further to understanding the role of uNK cells, this article discusses the proposed therapeutic approaches targeting uNK cells, aiming to provide a solution to RIF and RM patients avoiding IVF overuse and futile IVF attempts. Furthermore, this review aspires to provide a critical analysis that will set the basis to form the right hypotheses mapping future research.

## 2. Material and Methods

A comprehensive review of the literature was performed in PubMed/Medline, Embase, and Cochrane Central databases up to August 2021. A literature screening was performed, employing a combination of medical subject headings (MeSH) terms and keywords, including: “natura killer cells”; “NK cells”; “uterine naturall killer cells”; “uNK”; “implantation”; “implantation failure”; “recurrent implantation failure”; “RIF”; “IVF failure”; “miscarriages”; “pregnancy loss”; “recurrent pregnancy loss”; “RPL”; “recurrent miscarriages”; “RM”; “recurrent abortion”. The search was limited to full-length manuscripts published in English in international peer-reviewed journals. Original research articles describing studies performed mainly in humans as well as review papers were sourced. In order to provide an all-inclusive analysis of the current evidence, no specific inclusion and exclusion criteria regarding study selection process were employed. Regarding type of study, different types of studies were considered eligible to be included in this narrative review, namely prospective and retrospective observational as well as interventional studies and randomized controlled trials. Data originating from systematic reviews and meta-analyses are also presented. From the articles retrieved in the first round of search, additional references were identified by manual citation mining. Following literature assessment, a critical analysis with regard to the role of uNK cells on the pathophysiology of RIF and RM was performed. The authors further analyzed the current evidence with regard to the suggested therapeutic and management strategies of the RIF and RM patients.

## 3. The Role of uNK Cells in Reproductive Physiology and Pathophysiology

### 3.1. The Origin and Localization of uNK

Prior to discussing the role of uNK cells in the pathologies of RIF and RM, certain aspects of physiology should be put into perspective. Besides the hypothesis that uNK cells are derivatives of stem cell precursors in the bone marrow, the factors and mechanisms pertained to their accumulation in the endometrium are unclear [19]. The fact that a precursor cell type migrates into the endometrium to subsequently differentiate to a uNK cell is supported by evidence showcasing that a subset of NK cells in the peripheral blood expresses a similar antigenic phenotype to uNK cells [20,21]. Interestingly, uNK cells are either scattered in the endometrium or aggregated around spiral arteries and glands. Their perivascular location could be translated as an indicator suggesting the hypothesis of uNK cells’ migration from the peripheral circulation to the endometrium. On another note, this localization of uNK cells could also demonstrate their role in stromal decidualization and remodeling of the spiral arteries. The perivascular distribution of uNK cells has been considered to be precipitated by the diffusion of progesterone into the perivascular tissues [22,23]. Their increased endometrial levels during decidualization and implantation have been attributed to several reasons. Their ability to self-renew in situ accompanied by their intense proliferation capacity could present as a plausible explanation. Moreover, the upregulation of genes responsible for regulating proliferation during the luteal phase could also pose as another reason, while hormonal signals from the reproductive tissues seem to further support the interpretation of why an excessive NK cells number is observed in the endometrium [23,24,25,26].

Despite the great advances noted in the field, recent studies provide evidence indicating that the exact origin of uNK cells still merits further investigation. The hypothesis suggesting that uNK cells originate from hematopoietic precursor cells (HPCs), hosting in endometrium, is supported by experiments in which HPCs were successfully isolated from non-pregnant endometrium and early pregnancy decidua [27,28,29,30]. These isolated HPCs were successfully differentiated in CD56 ^bright^CD16 ^−^CD9 ^+^ uNK-like cells, when these were cultured in decidual stromal cell conditioned medium amplified with a cocktail of different cytokines [28,29,30]. Moreover, data provided from animal studies support the hypothesis of local uNK differentiation from HPCs. More specifically, researchers were able to isolate CD56 ^+^ uNK cells from non-obese diabetic/severe combined immunodeficiency/γC null immunodeficient mice, in which human proliferative endometrium was transplanted [31]. However, there are studies performed in non-pregnant endometrium reporting failure to isolate HPCs from human endometrium [32]. Contrarily, another interesting hypothesis supports that uNK cells could originate from differentiated peripheral blood NK cells, attracted to the endometrium from locally secreted cytokines and chemokines. This hypothesis is supported by studies highlighting that peripheral blood NK cells could successfully be transformed to uNK-like cells following culture in decidual stromal cell conditioned medium [4]. The same effect was also noted when peripheral blood NK cells were cultured in media supplemented with cytokines, including TGF-β or in hypoxic conditions [33]. Considering this, more studies are needed in order to draw safe conclusions regarding uNK cells’ origin. It is important to concur and reach a conclusion on the origin of uNK cells in order to provide the basis for designing appropriate treatment in cases where impaired uNK cells’ functionality is observed. In order to achieve this, standard procedures for uNK isolation and characterization should be in place.

### 3.2. The Role of uNK Cells in Implantation and Pregnancy

The influx of uNK cell count coincides with the phenomenon of implantation and the early stages of placentation, indicating that their role in these events is essential [34]. It is speculated that the route via which uNK cells regulate implantation success is controlling the trophoblast cell growth and activity. For an embryo to implant, attachment and penetration into the luminal epithelium of the endometrium is required. The most crucial aspect of this event is invasion of the trophoblastic cells in the decidualized stromal cells and their subsequent differentiation to numerous cell types [35,36]. Furthermore, the endovascular trophoblast is responsible for remodeling of the uterine spiral arteries from thick-walled vessels of musculoelastic properties to dilated tubes [35,36]. Spiral arteries constitute the main blood vessels of the endometrium, which are regenerated in each menstrual cycle with the involvement of uNK cells. During the early stages of a pregnancy, uNK cells are aggregated in close proximity to the spiral arteries and arterioles, indicating their role in controlling vascular changes [37]. It has been demonstrated that uNK cells surrounding unremodeled spiral arteries during the stages of early pregnancy could secrete Ang-1, Ang-2, VEGF-C, INF-γ, and matrix metalloproteinases, which, in turn, result to alterations in the vascular smooth muscle cells. These alterations—referring to their shape and alignment—enable the extravillous trophoblast’s cells to penetrate the arteries with a subsequent colonization of the wall. It is suggested that the diffusion of vasoactive factors by the uNK cells acts as a primer in the superficial spiral arteries facilitating trophoblast’s invasion [38].

Valuable data have emerged while investigating the interaction between uNK cells and the trophoblast. It has been indicated that uNK cells in humans could both enhance and inhibit the invasion of the trophoblast. Nonetheless, in rats and mice, it has been demonstrated that uNK cells’ role is to suppress the motility of the trophoblast. In these animal models, trophoblast invasion coincided with a state of diminished number of uNK cells [39]. Interestingly, in uNK cell-deficient mice, the trophoblast invasion was accelerated. Therefore, it becomes evident that the deficient number and/or function of the uNK cells is associated with impaired events of trophoblast invasion. The mechanism entailed in the interaction of uNK cells and the trophoblast is based on increased oxygen tension, which is established at the maternal–fetal interface. In the placentation site, the uNK cell depletion results to a decreased oxygen tension. This state is considered to prompt trophoblast’s differentiation to an invasive phenotype. Therefore, uNK cells seem to orchestrate placentation through a “hypoxia-sensitive adaptive reflex” [40]. More specifically, these complex functions of the uNK cells seem to play crucial roles for normal decidual angiogenesis. Uterine NK cells serve as master regulators of decidual angiogenesis and thus are able to control oxygen tensions at the maternal–fetal interface. It is well-established that these uNK cell actions are mediated by a cocktail of several angiogenic factors, such as vascular endothelial growth factor (VEGF), placental growth factor (PLGF), angiopoietin 1 (Ang1), and Ang2. These factors are produced by uNK cells during the initial stages of placentation [40,41,42,43]. Interestingly, it has been reported that both increased and decreased levels of decidual angiogenesis are associated with implantation failure and recurrent pregnancy loss in both humans and animal models [44,45,46]. The significance of these findings is highlighted by studies indicating that abnormal uNK sub-classes and/or increased uNK density could promote phenomena of increased angiogenesis. Increased angiogenesis, in turn, leads to increased peri-implantation blood flow, which possibly results in abnormal early maternal circulation and hence pregnancy failure due to excessive oxidative stress at the maternal–fetal interface [46]. Indeed, oxidative stress-induced placental dysfunction constitutes a common cause of the multifactorial and polygenic etiologies of recurrent pregnancy loss, defective embryogenesis, and implantation failure [47]. In summary, uNK cells control the trophoblast’s invasion through the regulation of oxygen tension at the maternal–fetal interface, which is attributed to the uNK cells’ ability to modulate angiogenesis at the intial stages of pregnancy. In the case of impaired function or abnormal uNK cells’ density, jeopardized angiogenesis, resulting in compromised trophoblast invasion, may occur. Moreover, in such cases, trophoblast apoptosis could be observed due to the excessive oxidative stress at the maternal–fetal interface.

On another note, the aforementioned angiogenic factors are secreted by the uNK cells in humans following the triggering and modulation of killer cell immunoglobulin-like receptors (KIR)/ human leukocyte antigen (HLA) interactions as well as the contribution of activating receptors, including NKp44, Nkp46, NKG2D, and NKp30. These recognition cell surface receptors interact with ligands and regulate certain cellular functions. HLA genes encode cell surface proteins, which play a role as a ligand for KIRs [48]. The decidual stromal cells express ligands for NKp30 and NKG2D, while the trophoblast expresses ligands for NKp44, suggesting that the uNK cell function is not only modulated through the trophoblast but also partially though interactions with the maternal tissue. What is more, expression of NKp30 and NKp44 splicing variants in the decidual environment has been proposed to play a role in reducing the cytotoxicity and modifying the secretion of cytokines in uNK cells. Furthermore, it has been suggested that the trophoblast expresses certain molecules, namely HLA-C, HLA-G, and HLA-E in the cell surface. In turn, they provide a protection against the cytotoxic function of decidual NK cells to the cytotrophoblast [49]. The recognition of fetal HLA-E by the decidual NK cells has been postulated to play a key role in the process of placentation. As demonstrated, HLA-E constitutes a ligand for the inhibitory receptor of NK cells CD94/NKG2A [50]. The interaction between HLA-E and the receptor instigates an inhibition of decidual NK cell’s cytotoxicity [51]. Trophoblast’s invasion unfolds due to events of motility and chemotaxis. The NK cells of the decidua enhance the trophoblast’s motility through the secretion of hepatocyte growth factor, while they control its chemoattraction to the remodeling site via the expression of certain chemokines, namely IL-8 and CXCL10. The presence of uNK cells has been correlated to a decreasing trophoblast invasion potential due to a molecular mechanism involving INF-γ [49]. What is more, based on in vivo studies, it has been postulated that the interaction between dNK cells and the trophoblast may be under the control of antigen-presenting dendritic cells which are detected in close proximity to the dNK cells during the first trimester [52]. Cytokines, growth factors, and hormones are further contributing to the establishment of this interaction. An interesting point has been raised in the literature, highlighting the fact that uNK cells are strictly located in the endometrium while the trophoblast invades deeper into the inner third of the myometrium where the absence of uNK cells has been described. Could this be a piece of evidence contradicting the involvement of uNK cells in implantation? The fact that—albeit not commonly—trophoblast invasion can occur even in extrauterine sites in cases of ectopic pregnancies where no uNK cells are detected further adds to the above concern [23]. Yet, the role of uNK cells in modulating the trophoblast differentiation and subsequent invasion has not been fully elucidated in humans [49].

### 3.3. Immunological Alterations Prompted by uNK during Implantation and Pregnancy

A prerequisite for the abovementioned events is for certain modifications regarding the immunological profile of the endometrial environment to be ensued. These modifications are imperative not only for a successful implantation but also to sustain a healthy pregnancy. It has been demonstrated that certain immune-related genes are upregulated in the endometrium during the window of implantation. These genes have been associated with stimulating proliferation of uNK cells, as well as inhibiting the cytolytic activity of uNK cells [26]. Therefore, their role in implantation by enabling an immune tolerance of the implanting embryo presents as a concrete hypothesis.

Pondering on the cytolytic nature of NK cells, one would expect that, in order for implantation to occur, a decrease in the number of uNK must be observed. However, the biological paradox of an influx of uNK cells during the events of implantation suggests that their “killer mode” is solely one side of the coin [26]. The cytolytic function of uNK cells isolated from early pregnancy decidua towards fetal cells is reduced, which has been attributed to the expression of human leukocyte antigen G (HLA-G) by placental trophoblast. HLA-G is a molecule considered to participate in the maternal immunological accommodation of the semi-allogeneic fetus to establish an interface between the mother and the fetus [53]. In a nutshell, HLA-G is a fetal molecule that exerts a crucial impact in the maternal immune response that may be compromised by the presence of excessive uNK cell levels.

Cytokines are essential contributing factors to a successful pregnancy. An accumulating body of evidence has proposed that the implantation process is under the control of growth factors and cytokines some of which are under steroid control [54]. Uterine NK cells secrete various cytokines and growth factors, namely IL-1β, IL-2, IL-4, IL-6, IL-8, IL-10, TGF-β1, and TNF-α, as well as numerous angiogenic growth factors, namely VEGF-A, VEGF-C, angiogenin, PGF, and keratinocyte growth factor [55,56,57]. Another crucial factor that promotes trophoblast invasion along with the spiral artery remodeling is proteolytic enzymes which are required for the extracellular matrix breakdown. Towards that goal, metalloproteinases MMP-1, MMP-2, MMP-7, MMP-9, and MMP-10, as well as tissue inhibitor of metalloproteinases are secreted by uNK cells [58].

### 3.4. uNK Cells in RIF and RM 

Little is known on the events prompting the phenomenon of excessive accumulation of uNK cells in certain cases of infertility. A speculation that an overall dysregulated endometrial environment potentially constitutes the basis for uNK aggregation has been voiced [59]. The association between the number of uNK cells and the onset of RIF and RM has been investigated by numerous studies. The vast majority of these studies performed endometrial biopsies and employed women with no history of infertility to serve as the control group. Immunocytochemistry techniques were applied, and subsequent analysis was conducted to detect the uNK cells in the acquired samples. Furthermore, risk factors such as obesity—a common finding for both RIF and RM cases—have been investigated in order to examine their association and impact to uNK cells’ biology. Maternal obesity seems to exert a detrimental impact to the reproductive outcome. Interestingly, obesity—presenting as a risk factor for RIF and RM—is accompanied by immunological alterations such as infiltration of immune cells and adipose tissue expansion [60]. Due to this compromised state, an alteration to the cytokine gene expression profile of uNK cells has been demonstrated in animal models [61], while the secretion of pro-inflammatory cytokines affects the uterine microenvironment [61,62]. It has been further reported that a high fat diet could result to a reduced expression of IFN-γ in uNK cells [63]. Therefore, obesity seems to hamper vascularization by limiting the ability of uNK cells to modulate artery remodeling.

#### 3.4.1. The Case of RIF Patients

The cases of RIF patients in the clinical practice of IVF have been in the spotlight of research in order to better comprehend the identity of this pathology. In these cases, recurrent events of implantation failure occur, despite the fact that good quality embryos are employed in the embryo transfer procedure, showcasing an underlying endometrial factor as the potential culprit. However, validating this speculation and decoding the endometrial molecular dialogues is hindered by the cyclical changes this tissue is subjected to during the menstrual cycle [19]. To elucidate the endometrial contribution in cases where implantation fails repeatedly, an investigation on the role of uNK cells has been pursued. An increased number of uNK cells has been identified in pre-pregnancy endometrial samples in one study including women with RIF following unstimulated IVF cycles [59]. Additional studies have indicated that a higher number of uNK cells in the preimplantation endometrium is expected in women with RIF [17,18]. The increased accumulation of uNK cells has been considered to be either a solid factor resulting in impairment of implantation or an indicator of an overall compromised endometrial environment hampering the embryo’s potential to implant. More ambiguous evidence is presented by other studies reporting that RIF patients presented with either a normal or an increased uNK cell count [54]. One study concluded that normal lymphocyte subpopulations were present in the endometrium of young women with a history of RIF who were subjected to IVF due to idiopathic infertility [64]. Interestingly, a systematic review published in 2011 proposed that any abnormality in uNK cell numbers is not indicative of the pregnancy outcome for women subjected to assisted reproduction [65]. Following the considerable limitations of the studies included in the aforementioned systematic analysis, the prognostic value of uNK cells count was disputed by the authors, as it lacks a concrete connection with a subsequent pregnancy outcome. Another systematic review and meta-analysis provided data indicate that both NK cell analysis and immune therapy targeting uNK cells should not be provided in routine clinical practice and should only be considered in the context of clinical research [66]. However, it is of paramount importance to investigate not only the number of uNK cells but also the biological activity and functionality of these cells in pathological cases, including RIF. Data provided from studies in the field indicates a higher proportion of CD56^dim^ NK cells in the peripheral blood of patients presenting with implantation failure. Moreover, experiments performed on in vitro cultured NK cells, isolated from the peripheral blood of patients presenting with RIF, indicated an increased NK cell cytotoxicity compared to both healthy multiparous and successful IVF control groups [67]. A summary of the current evidence with regard to the role of uNK cells in RIF is presented in Table 1. Considering the validity of these findings, one should note the high risk of bias characterizing these studies. On one hand, it is well established that peripheral blood NK cells cannot be safely employed to predict neither the number nor the biological activity of the uNK cells, as uNK constitute a unique uterine subpopulation. On the other hand, numerous parameters could affect both the number and the biological activity of the peripheral blood NK cells, including the biological diversity among the studies’ participants, and thus correlating peripheral blood NK cells profiling with the reproductive outcome may represent an extrapolation. Considering this, studies evaluating the biological functionality of uNK cells in patients presenting with RIF are limited, and thus no safe conclusions can be drawn. Even if uNK exert a significant role in RIF pathogenesis, the pathophysiological mechanism remains unknown.

#### 3.4.2. The Case of RM Patients

In patients with recurrent miscarriages (RM), the uNK cells’ endometrial profile is characterized by an elevated concentration of cytotoxic CD16(+) CD56^dim^ cells and decreased concentration of CD16(-) CD56^bright^ cells. The phenotype of CD16(-) CD56^bright^ is associated with the secretion of cytokines, namely macrophage-colony-stimulating factor (M-CSF) and granulocyte-macrophage-colony stimulating factor (GM-CSF), which are considered essential for placental growth [68]. Therefore, fetal loss may be caused by both uNK cells’ intense cytotoxic function as well as by the lack of adequate number of cytokines to support placental growth [69]. On the other hand, the concept that uNK cells may enable even abnormal blastocysts to implant, albeit ultimately resulting in miscarriage, has been proposed [70]. Interestingly, adding to the above speculation, data demonstrating that increased levels of uNK cells are detected in histological samples originating from miscarriages of chromosomally abnormal embryos compared to normal ones has emerged in the literature [71]. Numerous studies have indicated an association between an increased population of uNK cells in women experiencing recurrent miscarriages [72,73,74,75,76,77].

On the contrary, several studies indicating no correlation between the uNK cells count and RM pathology are published in the literature, showcasing that pre-pregnancy uNK cell count lacks the ability to predict the pregnancy outcome [68,78]. Employing flow cytometry, it has been reported that in RM patients CD16(-) CD56^bright^ NK cells were decreased, and CD16(+) CD56^dim^ NK cells were increased in the luteal phase endometrium [68]. A study performed in a limited number of patients by Quenby et al. indicated that enhanced levels of uNK cells were detected in women who miscarried in comparison to those who achieved a live birth [79]. Interesting data are also provided by a recently published prospective study investigating the expression of natural cytotoxicity receptors (NKp46, NKp44, and NKp30) and cytokine production (tumor necrosis factor-α and interferon-γ) on endometrial uNK cells in women with recurrent pregnancy loss (RPL) or implantation failure [80]. The percentages of NKp46^+^ cells were significantly lower in the RPL group as well as in pregnant individuals with a medical history of RPL in comparison to healthy participants. Additionally, the uNK cells, obtained from the RPL patients, expressed lower levels of tumor necrosis factor-α- and/or interferon-γ, indicating an abnormal uNK activity. These findings demonstrate the presence of abnormal uNK cell regulation in women with reproductive failure [80]. A summary of the current evidence with regard to the role of uNK cells in RM is presented in Table 2.

Hitherto, studies primarily showcase evidence indicating a certain pattern of increased uNK cells in patients with implantation and pregnancy failure [54,59,69]. This trail of thought leads to the formation of the hypothesis that perhaps this association indicates a causative relationship between increased levels of uNK and RM. It should be further emphasized that these observations have been validated in both artificial cycles employing stimulation and luteal phase support protocols as well as in natural cycles. As aptly commented by Laird, drawing conclusions on the association between uNK cell count and RM based on studies investigating miscarriage is questionable since the levels of uNK cells may not represent the cause of pregnancy loss but the result of it. Hence, this may be viewed as a catch-22 scenario. To add to the confounders entailed in these attempts to investigate the correlation between uNK cells and RM pathology, it has been voiced that the uNK cell count in RM patients could be affected by a potential previous birth since pregnancy and subsequent birth lead to changes regarding both the size and vascularization of the uterus [19]. Whether a molecular mechanism is involved in disrupting the establishment of implantation due to the harmful effect of uNK cells to the invading trophoblast remains a mystery. The contradictory data stemming from all these studies indicate that there is still insufficient evidence to enable drawing robust conclusions in regard to the role of uNK in these critical pathologies.

#### 3.4.3. Considerations Emerging while Critically Assessing Literature

**Figure 1:**
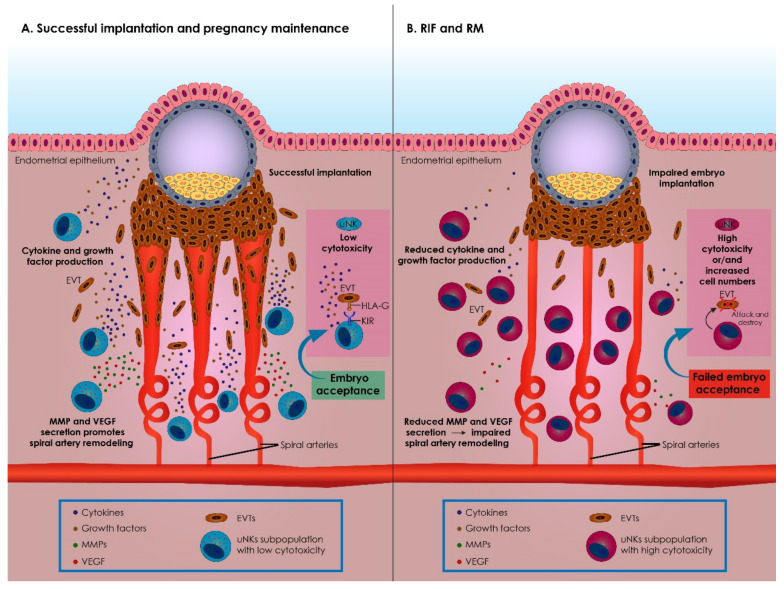
A summary of the role of uterine natural killer (uNK) cells on the events entailed in successful embryo implantation and maintenance of a pregnancy, as well as on the pathophysiological mechanisms involved on recurrent implantation failure (RIF) and recurrent miscarriage (RM), respectively. (**A**) Successful implantation and pregnancy maintenance. In physiological conditions, uNK subpopulations presenting with low cytotoxicity constitute the predominant leucocyte population in the decidua. During implantation, uNK cells interact with the extravillous trophoblast cells (EVTs), acknowledging the human leukocyte antigens G (HLA-G) via their killer cell immunoglobulin-like (KIR) receptors. These interactions are essential for several reasons. To begin with, these interactions lead to maternal immunological accommodation of the semi-allogeneic fetus, establishing an interface between the mother and the fetus. Additionally, these interactions trigger uNK cells to secrete several cytokines and growth hormones, promoting trophoblast invasion. Following their triggering, uNK cells secrete several matrix metalloproteinases (MMPs) and angiogenic factors, such as vascular endothelial growth factor (VEGF), regulating remodeling of the spiral arteries. Successful implementation of these events is essential for achieving implantation and pregnancy maintenance. In summary, uNK cells constitute master regulators of the events entailed during embryo immunological acceptance during EVTs invasion as well as during spiral arteries’ remodeling. (**B**) Events entailed in implantation failure leading to inadequate pregnancy maintenance in RIF and RM. When uNK cells present with increased numbers and/or with an abnormally increased cytotoxic phenotype, all the events entailed in normal embryo implantation are dysregulated. Due to their increased cytotoxic phenotype, this abnormal uNK cells subpopulation fails to appropriately interact with EVT cells, and instead they attack and destroy EVTs. Furthermore, secretion of growth hormones and cytokines by abnormal uNK cells is compromised, reducing EVTs invasion into the decidua. Ultimately, abnormal uNK cells fail to promote spiral arteries remodeling. These events lead to implantation failure or to impaired pregnancy maintenance, eventually resulting in miscarriage. RIF: recurrent implantation failure; RM: recurrent miscarriage; uNK: uterine natural killer cells; EVT: extravillous trophoblast; HLA-G: human leukocyte antigen G; KIR: killer cell immunoglobulin-like receptor; MMPs: matrix metalloproteinases; VEGF: vascular endothelial growth factor.

Assessing the results reported by studies that investigate the association between uNK cells and RIF or RM, discrepancies are revealed that should be extensively discussed. There is a vast heterogeneity amongst studies pertaining even to the definition they employ for RIF or RM patients. The deafening heterogeneity in the characteristics of the recruited patients may constitutes a significant confounder and justify contradictory results. What is more, prior to jumping to any conclusion in terms of the role of uNK cells in RIF or RM, it should be noted that there is striking controversy between researchers on what constitutes “elevated uNK levels”. Interestingly, even the definition of what constitutes “normal” has yet to be agreed on. Concurring on what should be evaluated as “a normal range” for uNK levels is challenging since by definition acquiring endometrial samples from healthy fertile patients presents with difficulties and limitations. Further to that, there is a lack of consensus on the evaluation methods employed for recording uNK cell numbers [77,81]. The proposed association between uNK cell numbers and RIF or RM cases has raised a demand for establishing an accurate and reliable protocol for assessing both peripheral blood NK and uNK cell numbers. Investigating current data on the potential causative relationship between uNK RIF and RM, the possibility that uNK dysregulation could contribute to RIF and RM emerges. In this case, assessing the degree of dysregulation may be of value. Nonetheless, it appears that perhaps it is not the degree of dysregulation that may drive events leading to RIF and RM but rather the timing this dysregulation occurs, along with the uNK cells’ density and the subtypes detected (Figure 1).

Prior to advocating the potential clinical application of uNK cells in diagnosis, prognosis, and treatment of patients with RIF and RM, a step forward should be taken in terms of introducing a standardized methodology for assessing the density of uNK cells, as mentioned above. Buttressing the fact that the discrepancies may constitute valid grounds for such contradicting evidence amongst studies, it has been reported that, in women with RIF, different subpopulations of lymphocytes may or may not be detected due to the methodology employed [59,64]. Flow cytometry or immunocytochemistry allow identification of uNK cells. Nonetheless, both present with confounders. For instance, in flow cytometry, agents from the peripheral blood could compromise the results, contrary to immunocytochemistry, where cells in the blood vessels may be easily distinguished [59]. This fact becomes relevant when we consider that the lack of a standardized method of evaluation is considered a hindrance for researchers when publishing uNK data. Resolving this lack of consensus regarding what is a normal or excessive uNK cell numbers is an imperative prerequisite that will ascertain publication of useful data by different sources. This will, in turn, enable a comparison between studies and safe conclusions to be drawn. Another limitation that published studies are posing refers to the variety of stimulation protocols to which women undergoing IVF have been subjected. NK cell subpopulations may undergo alterations associated with the stimulation protocols employed. Therefore, comparing studies that are conducted in IVF cycles employing different stimulation strategies raises a bias [69]. Studies investigating the efficiency of the aforementioned treatments and pharmaceutical protocols present with severe confounders stemming from the procedure of endometrial biopsy employed. Performing endometrial biopsy to evaluate a treatment’s effect on uNK cells’ levels may compromise assessment of the therapeutic effect itself [77]. It is imperative, albeit challenging, for studies to record uNK cells in peripheral blood simultaneously with the biopsy in order to elaborate on a potential systemic or local effect [74].

A summary of the role of uNK cells on the events entailed during embryo implantation as well as on the pathophysiological mechanisms involved in RIF and RM is presented in Figure 1.

## 4. Immunotherapy Options for uNK Related RIF and RM

There are several treatment protocols proposed in literature serving as tools for the management of patients diagnosed with RIF and RM that target the immunological background of these diseases. However, each case of RIF and RM may be attributed to a different underlying factor, not only adding another level of complexity but rendering establishment of an optimal practice consensus unrealistic. As expected, various approaches have been proposed and examined for patients with RIF and RM. However, concerns and considerations are raised with respect to the efficiency of these treatments due to the ambiguous evidence provided by literature. Hitherto, none of these proposed approaches have achieved clinical routine status application, and hence they retain their experimental status. The hypothesis that these treatments modulate uNK cell number but fail to improve adverse pregnancy outcomes should be raised. This may be indicative of additional mechanisms participating in the establishment and maintenance of pregnancy that probably require the contribution of cytokines, growth factors, and hormones to orchestrate the cross-talk between embryo and endometrium. Further data will delineate their value and establish or dispute immunotherapy approaches. This review examines proposed treatment options for uNK related RIF and RM.

### 4.1. Glucocorticoids 

As uNK cells express glucocorticoid and ER-β receptors, it has been claimed that steroids could be recommended as a treatment for RM [75,76]. Along these lines, prednisolone has been proposed as the glucocorticoid drug of choice as the placenta metabolizes it via 11 beta-hydroxysteroid dehydrogenase and the embryo’s drug exposure is kept to a minimum [76]. The use of prednisolone extending not only in cases of RM but also in cases of RIF has been reported in clinical practice. The hypothesis that the high number of uNK cells in the endometrium could be reduced with the administration of prednisolone has been confirmed by a study investigating the effect of prednisolone in 85 women with idiopathic RM [73]. It has been further indicated that administration of prednisolone exerts a positive effect on the endometrial immunological profile of approximately half the cases of RIF with over-immune activation [54]. A retrospective analysis of 164 women reporting recurrent reproductive failures including cases of both RM and RIF demonstrated a significant reduction in the uNK levels following prednisolone administration. Despite this observation, no improvement on pregnancy outcomes was reported [77]. On the same note, no significant difference concerning the live birth rate among women with RM that received prednisolone therapy has been observed compared to the placebo group in a randomized controlled trial (RCT) [76].

Nonetheless, these studies are accompanied by limitations such as the small sample size, rendering their conclusions on the effect of prednisolone therapy rather uncertain. Nonetheless, the odd case report may stand out, reporting on a patient with 10 previous miscarriages who was able to achieve a pregnancy following intrauterine prednisolone administration, which resulted in a subsequent live birth [82]. A low-dose prednisolone protocol prior to and after embryo transfer exerts no significant impact on pregnancy and implantation rates, as indicated by an RCT [83]. A meta-analysis was performed based on pregnancy outcomes following prednisolone treatment, concluding that prednisolone therapy improves pregnancy outcomes in women with idiopathic RM [84]. A study performed by Cooper et al., 2019 constitutes the sole attempt so far in literature to not only examine the efficiency of prednisolone in a well selected group of RIF patients, but further to present the pregnancy outcome following this treatment protocol. The findings suggest that, following prednisolone administration, a decrease in the uNK concentration could be observed. Nonetheless, this decrease did not ascertain a normal uNK concentration range for all patients. Despite the somewhat limited and not extensive mitigation of excessive uNK levels in the uterine environment, no improvement of pregnancy outcome was observed. It appears that, for both RIF and RM cases, there are contradictory evidence that either confirm or dispute the use of prednisolone toward managing uNK cells’ detrimental levels.

The mechanism responsible is elusive, however it is speculated that the glucocorticoid receptors of uNK cells may interact with prednisolone—a steroid—and the aftermath of this interaction remains to be elucidated. Studies suggesting no improvement in pregnancy rates following prednisolone treatment present with the limitation of late drug administration either at the time of oocyte retrieval or during embryo transfer [19]. The maintenance of pregnancy has been confirmed in women with RM following lymphocyte immunotherapy protocols [85]. Nonetheless, the alarming fact that recent studies find no evidence of a beneficial impact of prednisolone on pregnancy rates should be raised [77]. Concerns on efficiency of prednisolone should be addressed prior to offering this treatment routinely. Despite the inconclusive evidence on the efficiency of prednisolone in managing cases of RM and RIF, it is reassuring that its administration seems not to be accompanied by adverse effects on pregnancy, presumably due to its low fetal uptake. Concerning side-effects, mood alterations, namely euphoria and depression, have been documented, while increased appetite and mild epigastric discomfort have been further presented [73]. Insomnia, increased appetite, headache, hallucinations, palpitations, hirsutism, irritable bowel syndrome, and nausea have also been reported in a pilot randomized controlled trial with 160 participants [76]. Concerning pregnancy complications, no statistically significant difference was observed among women who received prednisolone treatment and the control group. In the same study, the incidence of one baby with small for gestational age (SGA) was documented [77]. Interestingly, a prospective controlled study examining pregnancy outcomes following exposure to corticosteroids during the first trimester showcased that no major teratogenic risk is entailed. Nonetheless, in utero exposure to glucocorticoids may be associated with a higher incidence of preterm births and low birth weight [86].

### 4.2. Intralipid Therapy

On the same note of immune modulating therapies, several studies have suggested the potential effect of intralipids in modulating the cytotoxicity of NK cells along with the secretion of pro-inflammatory cytokines [87,88,89]. Intralipid therapy is a 20% intravenous fat emulsion, which has been introduced in the medical practice for parenteral nutrition [90]. As a result, the implementation of intralipid therapy in order to mitigate the detrimental effects of elevated uNK cell count has been proposed in the literature. The molecular mechanism that enables intralipids to suppress NK function is elusive; however, an extrapolation on the already established knowledge on fatty acids may be of significance. Intralipid molecules act as ligands for the G-protein-coupled receptor that results in activating the cAMP signaling pathway that is associated with the NFkB pathway. The NFkB pathway ultimately modulates the transcription of the DNA and controls essential immune responses [89]. Several studies have suggested that intralipid therapy may be employed in cases of reproductive failure. Not only is the abnormal activity of NK cells modulated, but live birth rates appear enhanced following this therapeutic protocol [87,91]. Intralipids have been proposed to effectively reduce the enhanced NK activation and production of cytokines [87,91]. On the contrary, more recent data sourced by a double-blind randomized study indicated no increase in clinical pregnancy rates in patients with RM who were subjected to intralipid therapy [92]. No improvement in live birth rates in patients with RIF following intralipid therapy has been reported by Martini et al., in accordance with numerous studies that showcase no clinically significant impact of this therapeutic approach [93,94,95]. The lack of sufficient evidence to allow for safe conclusions to be drawn on the efficiency of intralipid therapy in women undergoing repeated implantation failures is clear. Large-scale studies are required to solve this conundrum prior to recommending it for routine use [93]. Thus far, recommendations that advise against routinely offering intralipid therapy for the treatment of RIF or RM have been voiced. The financial cost of intralipid administration outweighs their beneficial effect while failing to substantially enhance the live birth outcome [95]. Intralipids’ immunosuppressive properties should be further evaluated, since the research concerning their implementation in reproductive disorders has been limited. Safety concerns have been raised, and as suggested in several medical conditions, they include thrombophlebitis, dyspnea, nausea, hyperlipemia, and allergic reactions. Nonetheless, no side effects have been reported in cases of young women with reproductive failure [89]. Intralipids are considered a safe immunomodulatory agent with no reported adverse effects [92]. Reduced risk of teratogenesis and congenital abnormalities has been identified [96]. Large randomized controlled trials to determine the benefit of intralipids in the treatment of recurrent implantation failure or miscarriage are yet to be conducted [96].

### 4.3. Immunoglobulin

For the management of cases of RIF and RM, the administration of intravenous immunoglobulin (IVIg) has been also proposed. The mechanisms responsible for the modulation of NK cell functions by IVIg are obscure [97]. The rationale behind this line of strategy is based on the ability of IVIgs to mitigate the function of NK cells and promote alterations in the cytokine production. Therefore, IVIg is used as an “immunomodulatory” agent in an increasing number of immune and inflammatory disorders [98] by inhibiting the cell cytotoxic activity of NK cells in vitro and in vivo. This functional impairment of NK cells is accompanied by a spontaneous degranulation and IFNγ production that results in the exhaustion of the NK cell cytotoxic machinery [97,99]. It should be emphasized that IVIg administration is not recommended in cases of reproductive failure or recurrent pregnancy loss according to the UK Department of Health. The fact that it could interfere with Fc receptors in other immune cells, alter cytokine production, and neutralize complement components could severely compromise the maternal immune system [100]. IVIg infusion should be exercised with caution since anaphylactic reactions and renal insufficiency have been reported in high-dose IVIg treatments [101,102]. Fever, myalgia, headache, and fatigue constitute some of the mild side effects. In extremely severe cases, myocardial infarction, alopecia, thrombosis, hemolytic anemia, and aseptic meningitis are described [102,103]. Despite the long intimidating list of adverse effects, the occurrence of side effects is considered rare when the proper regimen of IVIg is administered. Data advocating the safe use of IVIg prior or during pregnancy for the fetus are still missing [102]. A meta-analysis along with a Cochrane review have reported no significant effect of IVIgs in patients with RM in terms of live birth rates [104,105], while several studies have highlighted a beneficial effect in decreasing elevated NK levels [106,107]. However, the abovementioned studies refer solely on observations on the peripheral blood NK cells. No evidence concerning implementation of IVIg therapy in mitigating the effects of uNK cells has been revealed in literature hitherto.

A summary of mechanisms of action, outcomes, and adverse effects regarding suggested immunotherapies for addressing uNK related RIF and RM is presented in Table 3.

## 5. Discussion

Human reproduction is truly an inefficient process [108]. The vast majority of failure occurs during the stages following fertilization suggesting that despite the advances and innovations in the techniques, strategies, and protocols to secure fertilization success, our poor understanding of the subsequent stages until implantation is what drives the low birth rates per cycle [109,110]. A key component in the process of embryo implantation is considered to be endometrial receptivity. Navigating possible pathways to shed light to the endometrial receptivity mystery, the unclear role of uNK cells has emerged as a promising field that merits investigation.

The reduced cytolytic activity of uNK cells, as well as their enhanced production of cytokines and angiogenic factors [23], are being advocated for their vital role in the establishment of a successful pregnancy outcome. This fact, coupled with the observation of fluctuating uNK levels during a menstrual cycle, indicates that uNK cells may be associated with endometrial receptivity dynamics and subsequent implantation potential [64]. As aptly emphasized, uNK cells’ characteristics are indicative of their protective as well as of their nurturing role during normal pregnancy [111]. The hypothesis that uNK cells participate in the complex phenomenon of hemi-allogeneic fetal graft tolerance emerged decades ago [112]. As evidenced in the literature, the gene expression profile of uNK cells includes immunomodulatory proteins such as integrins and inhibitory receptors in early pregnancy (KIRs). Their interaction with their HLA ligands is vital for regulating function and cytotoxicity [48]. This suggests their role in catering an immunosuppressive environment to secure an undisruptive mother–fetus dialogue [113]. The KIR gene complex demonstrates extensive polymorphisms, while the genes encoding these receptors form clusters in a region of the human genome. This cluster is considered to be amongst the most variant due to its gene content along with the sequence polymorphism [114]. The hypothesis that a “genetic variation in the repertoire” of KIRs is present in each individual [115] could set the tone for assuming that a certain KIR pattern could be a predisposal factor for the onset of pathologies such as RIF and RIM.

The association between uNK cells and RIF or RM cases has been extensively demonstrated by numerous studies as indicated by the review of literature herein. On the other hand, one should not fail to consider that data indicating the lack of connection between uNK cells and reproductive failure has also been reported. In a recent study, no correlation between elevated uNK levels and endometriosis was reported. Authors have further demonstrated the lack of statistical evidence that elevated uNK cells constitute a risk factor for the onset of RIF or RM [116]. These ambiguous conclusions call for further investigation. Prior to employing the suggested immunotherapies, namely glucocorticoids, intralipid, and IVIg infusion, to target the excessive uNK cell count, attempting to manage cases of RIF and RM definitive data are required. Proper dosage and concentrations, adequate number of infusions, ideal timing of the infusions, and implementation of these therapies in isolation or in combination are only a few of the parameters that should be elucidated. What is more, the longevity of treatment effects has yet to be determined. A comparison between the available therapeutic strategies should be performed to plan future research. For instance, it has been noted that the cost of IVIg therapy is estimated to be five times more than intralipid therapy. However, the severe adverse effects that accompany administration of IVIg serve as a limiting factor towards implementation [100]. Therefore, intralipid therapy has been proposed as a safer and more cost-effective strategy than IVIg for women experiencing RM [96].

Could targeting uNK cells with immunotherapy be considered a potential add-on treatment for restoring fertility and achieving pregnancy? Both their supplementary application in cases of reproductive failure and the lack of concrete evidence to support their effectiveness in enhancing live birth rates justifies them as experimental treatments. According to the Human Fertilization and Embryology Authority’s (HFEA) traffic light system classification, reproductive immunology tests and treatments are allocated a red signal since no RCT evidence to support their implementation is published. The traffic light system has been introduced by the HFEA to indicate the robustness of evidence available for each treatment that showcase their effectiveness in achieving pregnancy. Treatments options classified as red lack strong evidence but are not excluded from being offered in patients in a research set-up.

It should be further emphasized that this traffic light rating is only indicative of the efficiency and not the safety of the treatment option examined. Safety constitutes a multifaceted and perplexing issue, since a patient’s medical history dictates risk in a personalized manner. What is more, assessing the safety of these therapies should be conducted in the strict context of a specific fertility treatment. Therefore, it becomes evident that studies focusing on assessing the risks and the safety of immunotherapy targeting uNK cells should be designed and executed. Adopting such practices prematurely may set a dangerous precedent; therefore, health providers and patients should be equally alert and hesitant until robust evidence indicating enhanced reproductive outcomes along with safety emerge [100].

The prognostic value of uNK cells remains uncertain. Future validation of the role of uNK cells as a prognostic marker enabling prediction of RIF or RM has yet to be investigated, since a standardized quantification protocol is lacking [117]. Further to that, one could ponder, could there be a reference range/cut-off point of uNK cells number that may determine implantation potential and sustenance of pregnancy? Studying RIF, an elevated NK concentration has been reported in the peripheral blood of patients presenting with compromised embryo implantation dynamics following IVF [108,118]. These observations have opened the discussion on the potential role of circulating NK as a biomarker for early detection of reproductive failure. What is more, the potential of employing uNK cells as a more targeted biomarker for both diagnosis and individualized treatment is open to investigation. Since patients presenting with RIF and RM undergo a series of fertility tests to detect the underlying cause of infertility, endometrial biopsies may be performed to serve this purpose. Therefore, should it be deemed beneficial, an analysis on the individual’s profile of uNK cells could be performed by assessing cytotoxicity and uNK levels. However, the low-quality studies that have been performed hitherto are not facilitating any attempt towards achieving a robust biomarker status for either circulating or uNK cells. When the entailed molecular mechanisms are delineated, certain individually tailored for each patient therapeutic approaches may enhance the pregnancy outcome. Until then, measuring uNK cells and prescribing empirical immunotherapy in women with reproductive issues should be discouraged [65].

It becomes evident that the causative relationship between uNK cells and reproductive pathologies is still unclear. Vital questions have been proposed to guide future research aiming to elucidate the behavior of uNK cells and what triggers these cells to produce fetal growth promoting factors [119]. However, investigating and exploring uNK cells in depth is, by definition, hampered by the challenges in acquiring endometrial tissue samples during pregnancy, which could offer insight in the events of vascular remodeling. The fact that cells similar to uNK have been detected in species with no invasion of the trophoblast could suggest that more functions remain to be clarified [23]. Furthermore, little is known from a therapeutic point of view. In clinical practice, any type of excessive presence of lymphocyte-count benefits from an immune treatment protocol. Yet, although a decrease in uNK cell count seems to be achieved by these therapies, their true impact on reproductive outcome may be far from promising. The heterogeneous uNK cells population comprises numerous cell subsets with characteristics that are constantly being explored. Therefore, our understanding on the functionality of uNK cells is soon expected to extend beyond growth promotion and vascular modification [1]. What will the future hold, then, for uNK cells in the management of RIF and RM from diagnosis, prognosis, and treatment remains to be answered. It should be further noted that the molecular role of uNK cells in the reproductive system has not been in the spotlight of research in the past decade, which is evident by the lack of up-to-date novel basic research evidence in the literature. Future studies that will identify—from a molecular aspect to a tissue-specific level—the mechanisms involving uNK cells will shed light into their potential clinical value. Extrapolating conclusions based on studies that have been conducted in peripheral blood, NK cells fail to serve as an adequate basis to design future studies, since distinguishing these cells population featuring different phenotypical and functional characteristics is fundamental. Insight into uNK cells may provide a breakthrough in the assisted reproduction field enabling individualized therapeutic approaches based on the unique molecular interactions identified [120]. This relationship warrants further investigation and may institute a promising note in elucidating the complexity of the immunology of the maternal–fetal interface that echoes in numerous cases of idiopathic reproductive failure. 

In conclusion, there are discordant results and contradictory conclusions amongst scientific studies regarding the role of uNK cells on RIF and RM. These discrepancies arise from the heterogeneity of the studied population, along with the lack of standardized methodological protocols for evaluating both the normal range of uNK cell count, as well as their functionality. Considering the scientific insight that this study offers, it becomes clear that numerous aspects pertaining to the administration of these regimens as mentioned above should be put under the microscope when contemplating the possible therapeutic or diagnostic approaches. Key recent findings raise crucial questions that remain to be addressed prior to introducing uNK cells in clinical practice. How should the subsets of uNK cells be identified? Where are these subsets located throughout the course of the reproductive life? Is there a biological interpretation behind the diversity of phenotypes? [121] Routinely offering therapies targeting uNK cells fails to be justified by strong evidence, and recommendations that, respectively, advise against such practices should be taken into consideration. Future research is imperative to demonstrate the exact role of uNK cells in the pathophysiology of RIF and RM. Robust molecular data should drive clinical decision-making prior to proposing their use as a biomarker or targeting them employing therapeutic protocols to address uNK-related RIF and RM. This critical analysis review of the literature leads to formation of two specific questions. Firstly, is there a definitive causative relationship between uNK cells and RIF and RM, and if so, to what extent and through which mechanisms is this relationship driven? This is followed by the subsequent question focusing on how we can employ this knowledge to support an apt RIF and RM diagnosis, while contemplating RIF and RM management by therapeutically targeting uNK cells.

## Figures and Tables

**Table 1:** Summary of the current evidence with regard to the role of uterine natural killer cells in recurrent implantation failure, highlighting the examined parameters as well as the major findings of the analyzed studies.

Publication	Study Design	Study Group	Control Group	Interventions	Examined Parameters	Major Findings
[54]	Controlled clinical study	35 women with RIF after ET in IVF	12 fertile women	Ultrasound evaluation and endometrial biopsy on day 20	The balance between IL-12 and IL-18; the number of NK cells; and the vascular status	Distinct IL-12 and IL-18 patterns; significantly higher number of CD56 bright cells in patients with RIF
[17]	Pilot study	37 women with unexplained RIF following ET in IVF	8 fertile women	Ultrasonic evaluation and endometrial biopsy in luteal phase	Uterine artery Doppler; count of uterine CD56 bright cells/field; and quantification by real-time PCR to monitor IL-12 family, the IL-18 system (IL-18, IL-18R, IL18BP), and the IL-15 mRNA ratios.	Higher number of uNK CD56 bright cells/field in the preimplantation endometrium in RIF group; distinct IL-12/-15/-18 immune related mechanisms
[64]	Uncontrolled pilot study	10 young (30–35 years old) women with unexplained RIF following ET in IVF	Data obtained from the literature	Endometrial biopsy >6 months following the last IVF cycle	The number of CD56bright uNK cells	The percentage of the uNK subpopulation of CD56+CD16– and CD56bright CD16– cells did not differ between RIF patients and normal human endometrium
[59]	Prospective observational study	40 women with RIF	15 women with no history of infertility	Endometrial biopsy	The number of CD56+, CD16+, and CD69+ cells in the unstimulated endometrium of women with RIF	CD56+ cell density was significantly higher in the RIF group; there was no significant difference in the densities of CD16+ and CD69+ cells
[67]	Case-control study	20 women with IVF failure	Healthy control women: 36 normal multiparous women and 7 women with successful IVF	Peripheral blood sample collection; NK cell cytotoxicity level assessment via lactate dehydrogenase (LDH) release assay	compare the percentage of peripheral blood CD56(+) (CD56(dim) and CD56(bright)) cells and the level of NK cell cytotoxicity	The percentage of CD56(dim) cells and the level of peripheral blood NK cell cytotoxicity in women with IVF failure were significantly higher compared with the control group

**Table 2:** Summary of the current evidence with regard to the role of uterine natural killer cells in recurrent miscarriages, highlighting the examined parameters as well as the major findings of the studies.

Publication	Study Design	Study Group	Control Group	Interventions	Examined Parameters	Major Findings
[68]	Prospective study	Recurrent aborters	Normal	Endometrial biopsy in the secretory phase; assessment of endometrial leucocyte via two-color flow cytometric analysis	Immunophenotypic characteristics of endometrial leukocytes from nonpregnant recurrent aborters	Recurrent abortion group: lower levels of CD8+ T lymphocytes; increased CD4:CD8 ratio; increased proportion of CD20+ B leucocytes; increased CD16+CD56 dim uNK cells; and decreased CD16-CD56 bright uNK cell
[69]	Case-control	Abortion following IVF	Delivery following IVF	Peripheral blood samples to assess the expression of CD3, CD4, CD8, CD16, and CD56 using FACScan; analysis of NK cytotoxicity in blood sample via 51Cr Assay; endometrial samples to analyze the expression of CD16 and CD56 via FACScan	Expression levels of CD3, CD4, CD8, CD16, and CD56 in the peripheral blood; NK cytotoxicity in peripheral blood; expression levels of CD16 and CD56 in endometrial samples	Abortion group: higher levels of CD56+ and CD16+CD56+ cells in the peripheral blood on the day of ET; increased levels of CD16+CD56dim uNK cells and decreased levels of CD56bright uNK cells in endometrial samples
[71]	Case-control	Chromosomally normal abortion; chromosomally abnormal abortion	Selective termination of normal pregnancy	Peripheral blood samples as well as villi and decidual samples were samples	NK cell profile in peripheral blood samples as well as in decidual samples	Chromosomally normal abortions: lower levels of the decidual CD56+16-uNK cells; no difference regarding decidual CD56+16+ uNK cells
[72]	Case-control	29 women with recurrent miscarriages	10 parous women	Endometrial biopsies obtained in the luteal phase between days 7–10 following mid-cycle	Evaluation of the endometrial CD56+ cells	Increased mean numbers of CD56+ cells were documented in the endometrium of women with early RM
[79]	Prospective observational study	22 patients with idiopathic recurrent miscarriage	9 women with normal obstetric history	Mid-luteal phase endometrial biopsies	Profiling of endometrial leucocyte sub-populations	Higher number of CD4(+), CD8(+), CD14(+), CD16(+), and CD56(+) leukocytes in the RM group
[78]	Prospective observational study	17 women with RM	15 cases with male factor infertility	Endometrial sample collection during the peri-implantation period before subsequent pregnancy	Evaluation of natural killer (NK) cell markers, CD56 and CD16, a B-cell marker CD20, T-cell markers CD3 and CD8, and a specific T-helper (Th)2 and T-cytotoxic (Tc)2 marker	No significant difference in lymphocyte subset numbers or ratios was noted between the groups
[19]	A before and after study	29 women with RM	18 women attending for sterilization	Endometrial samples obtained on day 21+/-2; 20 mg oral prednisolone daily from day 1 to 21 of their menstrual cycle	Comparison of the percentage of stromal cells that were uNK between the groups prior and following prednisolone treatment of the RM group	Women with RM had significantly more uNK than the controls; prednisolone treatment significantly reduced the number of CD56 cells in the endometrium
[74]	Retrospective study	87 women with unexplained RM	10 normal control women	Biopsies obtained on days LH + 7 to LH + 9	Comparison of uNK cell number between the two groups; comparison of uNK cell numbers between RM individuals achieving live-birth vs. experiencing miscarriage in a subsequent pregnancy	The number of uNK cells in the RM group was significantly higher than in the control women; no difference was observed in uNK numbers between 19 women who miscarried and 32 women who had a live-birth in a subsequent pregnancy
[7]	Prospective study	28 women with recurrent pregnancy loss (RPL), 34 women with previous implantation failure	74 healthy women	Endometrial uNK cells were obtained from the mid-secretory endometrium prior to infertility treatment; blood sampled collected at 12, 20, 28, and 36 gestational weeks (GW) from pregnant women with and without a history of RPL	Expression levels of natural cytotoxicity receptors (NCRs) (NKp46, NKp44, and NKp30) and cytokine production in NK cells derived from the uterine endometrium of women with RPL; expression levels of NCRs in peripheral blood NK cells in pregnant women with and without a history of RPL	The percentages of NKp46+ NK cells were significantly lower in both women with RPL and pregnant women with a history of RPL; the percentages of tumor necrosis factor-α- and/or interferon-γ-producing uterine endometrial NK cells were significantly lower in women with RPL compared with controls

**Table 3:** Suggested immunotherapies for addressing uNK related RIF and RM.

Immunotherapies	Mechanisms of Action	Outcomes	Adverse Effects
**Glucocorticoids**	Regulate uNK cells’ proliferation and functionality via uNK cells’ glucocorticoid receptors	Decreased uNK cell numbers in endometrium	No pregnancy complications have been reported
	Reduce abnormal high uNK cell numbers	Positive effect on endometrial immunological profile	*In utero* exposure to glucocorticoids may be associated with a higher incidence of preterm births and low birth weight
	Reduce abnormal high uNK cytotoxicity	No established beneficial effect on pregnancy outcomes	Mood alterations, headache, nausea
**Intralipid therapy**	Intralipid molecules act as ligands for the G-protein-coupled receptor that results in activating the cAMP signaling pathway that is associated with the NFkB pathway	Modulates abnormal uNK activity	No side effects have been reported in cases of young women with reproductive failure
	NFkB pathway ultimately modulates transcription of DNA and controls essential immune responses	Trigger uNK cells’ cytokine secretion	Reduced risk of teratogenesis and congenital abnormalities has been reported
	Reduce abnormal high uNK cytotoxicity	No established beneficial effect on pregnancy outcomes	Risk for thrombophlebitis, dyspnea, nausea, hyperlipemia, and allergic reactions
**Intravenous immunoglobulin (IVIg)**	IVIgs mitigate the function of NK cells and promote alterations in the cytokine production	IVIgs decrease elevated NK levels in peripheral blood	Data advocating safe use of IVIg prior to or during pregnancy are still missing
	IVIgs inhibit cytotoxic activity of NK cells both in vitro and in vivo	No evidence concerning implementation of IVIg therapy in mitigating the effects of uNK cells has been reported	Anaphylactic reactions and renal insufficiency have been reported in high-dose IVIg treatments
	IVIgs induce spontaneous degranulation of NK cells and promote IFNγ production that results in the exhaustion of the NK cell cytotoxic machinery	No significant effect of IVIgs in patients with RM in terms of live birth rate has been reported	Fever, myalgia, headache, fatigue constitute some of the mild side effects. In extremely severe cases, myocardial infarction, alopecia, thrombosis, hemolytic anemia, and aseptic meningitis have been reported
